# Supplementary material for: Current practice in the management of new-onset atrial fibrillation in critically ill patients: a UK-wide survey
Source: PeerJ. 2017 Sep 8;5:e3716. doi: 10.7717/peerj.3716 (PMC5592903; doi:10.7717/peerj.3716)
Supplement: Supplemental Information 1 — Questions of online survey about critically ill patients developing new-onset AF. [file peerj-05-3716-s002.docx]

Current practice in management of new-onset atrial fibrillation (AF) in critically ill patients

Background:

New-onset atrial fibrillation (AF) is a common problem in critically ill patients and adversely affects outcome. Despite the high incidence of new-onset AF in the general ICU population, evidence for its treatment is limited. Guidance on management of atrial fibrillation is based on evidence obtained in patients outside the Intensive Care Unit. In this survey we would like to explore current UK practice for treatment of critically ill patients developing new onset fast atrial fibrillation.

The study questionnaire consists of two domains.

1. Recording of background and demographic data.

2. Identification of current treatment strategies for critically ill patients with new onset fast atrial fibrillation (heart rate >100/min)

Instructions:

Please complete the questions in sequential order.

- The survey software will enable or disable some questions depending on your responses.

- Based on validation testing we estimate it will take no more than 10 minutes to complete the survey.

- Please answer all questions as accurately as possible.

- Although for brevity we use the terms “ICU”, "intensive care" and “critical care” interchangeably, please also consider in your responses all high-dependency beds for which your service is responsible.

-We define “new onset fast atrial fibrillation” as atrial fibrillation with a heart rate of more than 100 beats per minute in patients without a previous history of atrial fibrillation.

Part 1: Background and demographics

Q1

Which of the following answers most accurately describes your hospital?

- District General Hospital

- Teaching Hospital

- Tertiary Referral Centre or University Hospital

Q2

How many patients are admitted to your Intensive Care Unit per year? (Please include level 2 admissions):

- <500

- 500-1000

- 1000-2000

- >2000

- I do not know

Q3

How many staffed Intensive Care beds (level 2 and level 3) does your Department have?

Q4

Please state the case mix of your ICU/HDU:

- Predominantly surgical

- Predominantly medical

- Mixed ICU

- Specialist ICU (please name specialty):

Q5

Please state your level of training:

- Consultant

- Trainee

- SAS

Q6

How many years of experience do you have in Critical Care?

- <1 year

- 1-3 years

- 3-5 years

- 5-10 years

- More than 10 years

Q7

Please state your complimentary specialty:

- Intensive Care Medicine only

- Anaesthesia

- Acute medicine

- Emergency Medicine

- Paediatrics

- Surgery

Part 2: Treatment strategies for new onset fast atrial fibrillation

Notes:   We define “new onset fast atrial fibrillation” as atrial fibrillation with a heart rate of more than 100 beats per minute in patients without a previous history of atrial fibrillation.

Q1

At which heart rate would you intervene in patients with fast AF and stable blood pressure?

- 100-119/beats per min

- 120-139/beats per min

- 140-159/beats per min

- >160/beats per min

- Independent of their heart rate I treat all patients who have developed new onset fast AF, even if the blood pressure remains stable

Q2

Which is your primary treatment goal in new onset fast atrial fibrillation in critically ill patients without a known cardiac history, in whom blood pressure remains stable?

- Rhythm control

- Rate control

- I have no primary treatment goal in these patients

Q3

Which is the most commonly used anti-arrhythmic drug for new onset fast atrial fibrillation in your ICU?

- Amiodarone

- ß-blocker

- Flecainide

- Diltiazem

- Digoxin

- Other anti-arrhythmics (e. g. Verapamil, sotalol): please specify:

Q4

In a patient with chest sepsis (no cardiac history, blood pressure 100/60 mmHg, receiving 15 ml/min (0.25 mcg/kg/min) noradrenaline), who develops fast new onset AF with a heart rate of 140-160 bpm, your primary treatment strategy consists of:

- Supplement electrolytes (magnesium and/or potassium) to a high normal level

- Supplement electrolytes to a high normal level and anti-arrhythmics

- Supplement electrolytes to a high normal level and DC cardioversion

- Anti-arrhythmics only

- DC cardioversion only

- DC cardioversion when anti-arrhythmics and electrolyte replacement fail to achieve rate and/or rhythm control

- I only intervene if blood pressure drops or inotrope requirements increase

Q5

In critically ill patients with new onset fast AF, which Serum Potassium level would you aim for?

- >3.5mmol/l

- >4 mmol/l

- >4.5 mmol/l

- >5 mmol/l

- I do not aim for a specific serum potassium level

Q6

In critically ill patients with new onset fast AF, which Serum Magnesium level would you aim for?

- 0.75-1 mmol/l

- 1.0-1.2 mmol/l

- >1.2 mmol/l

- I do not aim for a specific serum magnesium level

Q7

Which of the following represent the main reason(s) for your choice of anti-arrhythmics? Please tick all that apply.

- Availability from hospital pharmacy

- ICU drug policy

- Cost

- Pharmacokinetic advantages

- Pharmacodynamic properties

- Adverse effect profile

- Other, please specify:

Q8

When would you normally anti-coagulate critically ill patients with new-onset atrial fibrillation, if no contra-indications for anti-coagulation are known?

- New onset AF within 24 hours

- New onset AF within 48 hours

- New onset AF within 72 hours

- Before starting anti-arrhythmic medication

- After starting anti-arrhythmic medication

- Before DC cardioversion

- I do not regularly anti-coagulate critically ill patients with new-onset fast AF

Q9

Please tick all answers that reflect your views on stroke risk assessment in critically ill patients with new-onset atrial fibrillation?

- I do not use stroke risk scores routinely in critically ill patients with new onset AF to assess the need for anticoagulation

- I regularly calculate a risk score (e. g. CHAD2, CHA2DS2-VASc) to assess the need for anti-coagulation

- Stroke risk scores inaccurately reflect the risk of embolic events in critically ill patients with new-onset atrial fibrillation due to prothrombotic changes associated with critical illness

- Stroke risk scores favour anti-coagulation despite a higher risk of bleeding in critical illness

- Modified risk scores should be developed for critically ill patients with new-onset atrial fibrillation

Q10

In critically ill patients with new-onset fast atrial fibrillation which of the following do you consider appropriate for anticoagulation provided that no contra-indications are known? Please tick all answers that reflect your views.

- Intravenous High Molecular Weight heparin in therapeutic dose

- Subcutaneous Low Molecular Weight heparin in therapeutic dose

- Use of novel oral anti-coagulants (NOACs)

- Use of warfarin

- I do not regularly anti-coagulate critically ill patients with new onset fast AF

Q11

In critically ill patients with new-onset fast atrial fibrillation: (Please tick all that apply)

- I regularly perform or request transthoracic echocardiography

- I regularly perform or request transoesophageal echocardiography

- I do not routinely perform Echocardiography to guide treatment

- I only regularly perform Echocardiography in patients with cardiac history

Q12

Would you consider taking part in a clinical trial investigating treatment of new onset fast AF in the critically ill?

- Yes

- No

Q13

In the setting of a research study on general ICU patients, which anti-arrhythmic treatment would you primarily want to study as treatment for critically ill patients with new-onset fast AF?

- Amiodarone

- DC cardioversion

- ß-blocker

- Flecainide

- Diltiazem

- Digoxin

- Other anti-arrhythmics (e. g. verapamil, sotalol): Please specify:

Q14

In the setting of a research study investigating the effectiveness of anti-arrhythmics in critically ill patients with new-onset fast AF, would you accept a placebo arm, i. e. do you think it is acceptable to not treat new onset fast AF with anti-arrythmics as long as blood pressure and, if measured, cardiac output are maintained?

- Yes

- No
